# Debates in allergy medicine: food intolerance does exist

**DOI:** 10.1186/s40413-015-0087-7

**Published:** 2015-12-14

**Authors:** Y. Vandenplas

**Affiliations:** Department of Pediatrics, UZ Brussel, Vrije Universiteit Brussel, Laarbeeklaan 101, 1090 Brussels, Belgium

## Abstract

**Background:**

Health care professionals and patients mix and mingle (hyper)sensitivity, allergy and intolerance. The consequences are discrepancies which result in confusion. The following is a very personal point of view, intended to start a debate to come to consensus.

**Objectives:**

We aimed to clarify the proposed terminology for the primary health care professional from the point of view of the pediatric gastroenterologist.

**Results:**

Many patients present with symptoms “related to food ingestion”. We propose to use this wording if no underlying mechanism can be identified. Intolerance should be restricted to carbohydrate malabsorption causing symptoms. Allergy is restricted to IgE mediated allergy and non-IgE manifestations that can only be explained through an immune mediated mechanism, such as food induced atopic dermatitis and allergic colitis with blood in the stools. Unfortunately, primary heath care physicians have no diagnostic tools for non-IgE mediated allergy. A positive challenge test is a proof of a food-induced symptom, but does not proof that the immune system is involved. (Hyper)sensitivity suggests immune mediated mechanisms and should therefore not be used. The pathophysiologic mechanism of many food-related symptoms is unclear. The same symptom can be caused by allergy or be considered functional, such as infantile colic, gastro-esophageal reflux and constipation related to cow’s milk ingestion in infants. In fact, “functional” is used if the pathophysiologic mechanism causing the symptom cannot be explained. Since the long term outcome of “allergy” differs substantially from “functional symptom”, allergy should not be used inappropriate.

**Conclusion:**

“Food related symptom” should be used in each patient in which the pathophysiologic mechanism is not clear. Intolerance means a carbohydrate malabsorption that causes symptoms. Allergy should be used when the immune system is involved.

## Background

“Intolerance” is a term loaded with different meanings and interpretations. Intolerance, or hypersensitivity, relates to all reactions to foods, while allergy indicates that an immune mechanism is involved and atopy is the terminology for immunoglobulin E (IgE) mediated reactions. Intolerance is also used when the symptoms are related to carbohydrates, when the sugar is malabsorbed because of a deficiency of one of the disaccharidases (lactase, maltase, sucrase). In relation to clinical reactions to gluten, gluten intolerance is used as a synonym for celiac disease. According to the title of this paper, intolerance is used to describe a clinical reaction to food in the absence of an enzymatic deficiency and “easy to measure antibodies” such as in celiac disease or in IgE-mediated allergy. Indeed, there are patients who develop symptoms related to the ingestion of food and in who all the above tests are within normal ranges (Table 1). The mechanisms creating these symptoms is largely unknown. A “toxic effect” of milk like in pulmonary haemorrhage would be one possibility, allergic reaction mediated by yet to be identified mechanisms are possibilities; a totally different -but unknown- mechanism is as well a plausible hypothesis. Some patients do have positive (double blind) challenges although no underlying mechanism causing the symptoms can be identified.

**Table 1:** Proposed terminology

• Hypersensitivity: a reaction to an ingested food, whatever the mechanism causing the symptoms
• Food allergy: a reaction to a food that can be explained by an immune mediated mechanisms, IgE-mediated or non-IgE mediated
• Intolerance: a reaction to a food that is caused by a deficiency of a disaccharidase
• Food related symptom: any symptom that is/seems caused by a food but for which no causing mechanism can be indicated

## Cow milk related symptom as example

Let’s discuss cow milk (CM) as an example. Cow milk allergy (CMA) is a reproducible clinically abnormal reaction to a CM protein due to the interaction between one or more food proteins and one or more immune mechanisms [1]. CMA is the most frequent cause of food allergy and of allergic disease in infants, said to occur in about 0.5 % of exclusively breast-fed infants and between 2.0 to 5.0 % in CMP formula fed infants [2]. This range in reported prevalence is mainly due to selection of patients and differences in definition. According to a recent meta-analysis the self-reported lifetime prevalence of cow’s milk protein allergy (CMPA) is 6.0 % (5.7-6.4) and the food-challenge-defined CMPA prevalence was 0.6 % (0.5-0.8) [3]. Earlier frequency-reports have landed at 2–3 % incidence in the first year of life. In the Netherlands, 7 % of children visit the general practitioner because of symptoms considered to be related to cow’s milk [4]. The parental burden of these symptoms is also important. Mothers often perceive infants with unspecific symptoms suggestive of GI-CMPA as demanding and temperamentally difficult [5].

However, gastro-intestinal (GI) symptoms related to CM intake are estimated to occur in 10 to 15 % of formula fed infants [6]. Functional GI disorders occur in up to 50 % of infants, each accounting for 20 to 25 % [6]. Functional GI disorders in infants include a variable combination of often age-dependent, chronic or recurrent symptoms not explained by structural or biochemical abnormalities.

The gold standard to diagnose food allergy and CMA more specific consists of the “elimination-challenge” principle: the symptom(s) disappear if the offending allergen is retrieved from the diet, and symptom(s) re-appear when the food is re-introduced in the diet [2]. The challenge test is by preference performed double blind: the patient or parents and the physician evaluating the result of the challenge should not know if placebo or CM was tested. However, a positive challenge does not prove that there is an immune mechanism involved: a reaction during a challenge test only shows that the ingestion of the food is initiating symptoms.

Symptoms and signs caused by a hypersensitive reaction to CMP can be of allergic origin. Symptoms occurring relatively fast after ingestion of CMP are often accompanied by raised levels of specific IgE and/or positive skin prick tests. If these symptoms disappear under an elimination diet and relapse when CM is reintroduced in the diet, the diagnosis of “IgE-mediated CMA” can be considered to be established. Although false negative increased levels of specific IgE do exist, this occurs relatively seldom. Typical IgE mediated symptoms include urticaria, angioedema, vomiting, diarrhea and anaphylaxis. Dermatitis and rhinitis can be IgE and non-IgE mediated. Vomiting, constipation, hemosiderosis, malabsorption, villous atrophy, eosinophilic proctocolitis, enterocolitis and eosinophilic esophagitis are non-IgE mediated reactions. In addition, respiratory symptoms such as chronic rhinitis and asthma may be caused by CMA. Irritability, fuzziness and colic are sometimes the only symptoms of CMA.

Non-IgE mediated allergy is the only way how to explain efficacy of a CM elimination diet in infants with atopic dermatitis and negative (specific) IgE or skin prick test. Symptoms such as atopic dermatitis, respiratory symptoms such as chronic cough and wheezing, colitis with the presence of red blood in the feces, etc. can only be explained by (most of the time) non-IgE immune mediated reactions if they disappear during elimination and relapse during challenge. Sometimes indirect arguments for an allergic reaction such as eosinophilia or eosinophilic infiltration in colonic biopsies or a positive patch test can be found. More sophisticated tests do exist to demonstrate the involvement of the immune system, but these tests are not routinely available and are not of help to diagnosis and management in primary health care.

The interpretation of other GI symptoms such as regurgitation, vomiting, gastro-esophageal reflux (GER), constipation, diarrhea and infantile colic is more complex. Each of these symptoms has multiple etiologies, but each one has also been reported to be caused by (non-IgE mediated) CMA.

An extensively hydrolysed formula (eHF) may reduce regurgitation and GER because of a faster gastric emptying [7]. An eHF has also been shown to result increase defecation frequency and to decrease consistency. Partial and extensive hydrolysates have been shown in randomized controlled trials to decrease regurgitation, constipation and colic. Soy and even rice milk may be effective when constipation is caused by cow’s milk [8]. An eHF is recommended in infantile colic when allergy is clinically suspected [7]. However, many eHFs do have a reduced or absent lactose content, what also has been shown to reduce infant crying.

In relation to CM, the term “intolerance” should be restricted to carbohydrate maladsorption. Primary lactose intolerance does hardly exist in infants younger than one year. Whenever lactose intolerance occurs in young children, it is secondary to diseases such as celiac disease and GI infection(s), but it can also be caused by CMA. Hypersensitivity to CM (or another food) is often used to indicate increased IgG4 levels. Whether this is clinically meaningful or not remains controversial. Hypersensitivity differs from allergy as the immune system has not been shown to have a causal role.

## Long term outcome

It is important to not mix “CMA” with “functional GI CM-related symptoms” because the middle to long term outcome of CMA versus functional GI disorders differs substantially. The onset of allergic disease at a young age (as is the case with CMA) will predispose the child to a two times increased risk of developing asthma and allergic rhinitis later in life [2, 6]. Once the diagnosis of CMA is made after a positive challenge test, it is recommendation to put the child on an exclusion diet for 6 to 9 months, or up to the age of year if that is reached first [2]. (The higher the IgE levels, the longer CMA allergy will persist.) Although up to 90 % of the CMA children are CM tolerant by the age of three years, this is only the case in 50 % at the age of one year. Functional GI symptoms tend to improve much faster. Infantile colic and crying starts to decrease by the age of three to four months [7]. The frequency of regurgitation drops sharp from the age of six months onwards [7]. Functional constipation, on the contrary, does not tend to disappear spontaneously over time [8]. Thus, prognosis and long-term outcome differ for allergy or a functional GI disorder. One should not overlook that any of these manifestations can as well be caused by organic disease, different from allergy. This is the area where “CMA” versus “symptoms related to CM” becomes relevant. In function of the presenting symptoms, interpretation and therapeutic attitude may differ, influenced by the presenting symptom. A score, the Cow’s Milk related Symptom Score (CoMiSS), was recently developed to raise awareness of health care professionals for this entity [9].

Focussing on gluten, the situation is even more complex. The diagnostic criteria for celiac disease are well established. Other individuals may suffer from IgE mediated gluten allergy. But, there are patients claiming to develop symptoms when ingesting gluten and to feel much better on a gluten free diet. Whether this is non-IgE mediated allergy or more psychological related as may occur in aversion is debated. A casein-free and gluten-free diet is very popular in autistic children, but we showed recently in a double-blind placebo controlled trial that a short challenge during seven days was not accompanied by an increase of the behavioural symptoms [10]. IgG4 antibodies are often measured and increased in these individuals. According to most opinion leaders the presence of these antibodies means that the immune system has been in contact with these food antigens, but not that there is an abnormal immunological reaction. The fact that patients report disappearance of symptoms is often supposed to be placebo-induced. Also for IgG4, CMA was well studied. IgG4 anti-betalactoglobulin levels failed to show a relation with CMA [11]. Nevertheless, patients do spend hundreds of Euro to obtain “pages” of IgG4 levels to several foods and food components, to which –at least according to the interpretation of these labs- the patient is allergic or reacting.

## Adverse reactions and perception

Perception plays a major role in many adverse reactions to foods. Many Western individuals will reaction with aversion by the idea to have to eat insects or dog. Thus, once we move outside the classic adverse reactions to food that can easily be explained as occurs in diseases like IgE mediated allergy and celiac disease, cultural background does have an important role, and the “pro and con debates” start. Non-IgE mediated allergy is an accepted entity. If the patient develops objective symptoms such as blood in the stools, a non-IgE mediated allergic colitis is an accepted diagnosis. However, the same diagnosis is not (well) accepted in case the symptoms are more subjective such as nausea, itching, fatigue, feeling unwell,… Although the patch-test may play a role in these situations, its diagnostic accuracy has been insufficiently validated to be recommended [12]. Unfortunately, data from double-blind placebo controlled challenges are very limited in these situations; this is mainly due to the fact that patients refuse these. However, a positive challenge is not a proof of involvement of the immune system.

## Conclusion

Some patients develop symptoms related to ingestion of food that are “easy to classify” such as anaphylaxis, IgE mediated allergic symptoms, immune mediated disease such as in celiac disease. Lactose intolerance is another example. However, there are patients in who all standard diagnostic tests are within normal ranges. In order to avoid confusion, we prefer to designate these as “food ingestion related symptoms”.
